# Editorial: Advances in immunogenicity risk assessment, monitoring and mitigation of biologics

**DOI:** 10.3389/fimmu.2026.1979026

**Published:** 2026-09-30

**Authors:** Sophie Tourdot, Daniela Verthelyi, Marc Pallardy, Timothy Paul Hickling

**Affiliations:** 1 Pharmacokinetics, Dynamics and Metabolism, Pfizer Inc, Andover, MA, United States; 2 Thimmus, LLC, Potomac, MD, United States; 3 INSERM UMR 996, Universite Paris-Saclay, Orsay, France; 4 Quasor, Loughborough, United Kingdom

**Keywords:** AAPS, anti-drug antibodies (ADA), European Immunogenicity Platform, immunogenicity, immunogenicity mitigation, immunogenicity monitoring, immunogenicity risk assessment, new approach methodologies

## Introduction

The rapid evolution of biological therapeutics - ranging from classic monoclonal antibodies to increasingly complex engineered proteins, antibody-drug conjugates (ADCs), and therapeutic peptides - has revolutionized modern medicine. However, the potential of the host immune system to mount an unwanted immune response against these foreign agents remains a critical obstacle for their development. Anti-drug antibodies (ADAs) can alter pharmacokinetics (PK), compromise efficacy, or induce serious adverse events. This Research Topic, featuring 16 compelling articles, showcases how the field is shifting from reactive *post-hoc* clinical observation to proactive, model-guided prediction, advanced bioanalytical monitoring, and tailored mitigation strategies.

To guide readers through these advancements, we have structured this editorial around three interconnected pillars: (1) Non-clinical Immunogenicity Risk Assessment & Molecular De-risking, focusing on computational and *in vitro* tools to predict vulnerabilities early; (2) Novel Bioanalytical Assays, Modalities, and Standardization, highlighting how analytical platforms are adapting to complex formats and the need for harmonized monitoring; and (3) Clinical Translation, Safety Consequences, and Consensus Frameworks of computational and *in vitro* tools, connecting early predictions to real-world patient outcomes and landmark mitigation guidelines.

## Pillar 1: non-clinical immunogenicity risk assessment & molecular de-risking

Early-stage risk prediction is paramount to designing safer biotherapeutics. The articles in this section highlight how computational modeling and sophisticated *in vitro* assays are refining our ability to predict immunogenic liabilities. This is consistent with a broader industry effort to generate New Approach Methodologies (NAMs) for translational research.

Model-Informed Predictions: Rose et al. introduce a model-informed metric that transitions beyond predicting ADA incidence towards evaluating the potential impact of ADAs on therapeutic PK. This approach contributes to bridging the gap between early preclinical predictions and clinical outcome modeling.

Targeting Early Immune Events: Antigen internalization by antigen-presenting cells (APCs), particularly dendritic cells (DCs), is a rate-limiting step in T-cell priming. Lteif et al. provide a comprehensive perspective on how therapeutic antibody uptake by immune cells dictates immunogenicity. Implementing this science for drug development, Yin et al. present a high-throughput, real-time imaging approach to quantify DC internalization, offering a robust tool for early screening.

Sequence-Level Vulnerabilities: Qin et al. demonstrated that even minor sequence modifications, such as a single point mutation on the FLT3L-Fc protein, can significantly elevate immunogenicity risk, underscoring the criticality of *in silico* and *in vitro* de-risking.

Novel Mitigation Strategies: Jarvi et al. explore an immune regulatory adjuvant approach to mitigate the immunogenicity of monoclonal antibodies, presenting a proactive strategy to control unwanted immune responses from the outset.

## Pillar 2: novel bioanalytical assays, modalities, and standardization

As therapeutic modalities diversify, bioanalytical platforms must adapt to capture immunogenicity profiles accurately, while the field strives for industry-wide harmonization.

Standardization in Monitoring: A major hurdle in therapeutic drug monitoring is assay comparability. Wadhwa et al. detail the development of the first WHO reference panel for Infliximab anti-drug antibodies, a crucial step toward harmonizing clinical monitoring.

Platform Innovations for Complex Formats: Fu et al. developed a high-throughput, Gyrolab-based generic ADA assay to support antibody-drug conjugate (ADC) evaluation, optimizing sensitivity and throughput for non-clinical safety studies. Similarly, Valentine et al. propose a risk-driven, 1-tiered testing strategy for vidutolimod, streamlining the drug and anti-drug antibody testing process.

Addressing Modality-Specific Challenges: Puig and Shubow provide a comprehensive perspective on the immunogenicity of therapeutic peptides, focusing on product-related risk factors. For gene therapies, Chen et al. offer an opinion on the AAV2-associated liver injury, discussing the complex interplay of viral vectors and pre-existing immunity.

## Pillar 3: clinical translation, safety consequences, and consensus frameworks

The ultimate test of any immunogenicity risk assessment is its translation to the clinic, ensuring that findings directly inform patient safety and management.

Decoupling ADAs from Clinical Adverse Events: In a clinical evaluation of adalimumab, Tourdot et al. demonstrated that treatment-emergent ADAs do not necessarily correlate with immunologically related adverse events. Conversely, van de Beek et al. show that the TNFα binding domain of adalimumab elicits CD4+ T-cell responses in rheumatoid arthritis patients, highlighting the complexity of clinical responses (van de Beek et al.).

Clinical Context and Patient Factors: The immunogenicity of biotherapeutics can be highly context-dependent. Irvine et al. discuss this in the case of anti-SARS-CoV-2 antibody therapies, showing how immune complexes form during co-administration with mRNA vaccines, while Tsai et al. offer a commentary on the immunogenicity of dupilumab in adult versus pediatric patients.

Consensus for Tailored Mitigation: Representing major milestones, two articles from the European Immunogenicity Platform (EIP) are featured. Grudzinska-Goebel et al. deliver a harmonized, practical framework for overall immunogenicity risk assessment. Complementing this, Tourdot et al. provide best practices for T cell assays in non-clinical risk assessment, a critical set of NAMs for mechanistic understanding and translatability of the immune cells that significantly contribute to immunogenicity.

## Conclusion and outlook

The 16 contributions encompassing this Research Topic collectively demonstrate a pivotal paradigm shift in the field of biotherapeutics: the transition from reactive, *post-hoc* immunogenicity management to a proactive, predictive, and structurally harmonized approach. By integrating sophisticated *in silico* and *in vitro* de-risking models early in the pipeline, developers can now identify and engineer out potential molecular vulnerabilities before they ever reach the clinic. Furthermore, advancements in high-throughput bioanalytics and standardization, such as the first WHO reference panel for infliximab, ensure that once a drug enters clinical trials, the monitoring is robust, reproducible, and clinically meaningful.

Looking to the future, the immunogenicity landscape will only grow more complex as novel modalities, such as multi-specific antibodies, gene therapies, and engineered cell therapies, enter the mainstream. The outlook for the field relies heavily on three key frontiers:

1. Integration of Artificial Intelligence (AI) and Machine Learning: Expanding model-informed metrics to predict not just ADA incidence, but complex PK/PD impacts and clinical safety outcomes.
2. Personalized Immunogenicity Mitigation: Leveraging patient-specific factors (e.g., HLA typing, pre-existing immunity, disease state) to tailor bioanalytical monitoring and tolerance-inducing regimens.
3. Global Harmonization: Continuing the momentum built by consortia like the European Immunogenicity Platform (EIP) and AAPS to ensure that regulatory frameworks globally adopt unified, risk-based strategies.

Ultimately, mitigating immunogenicity is no longer just a regulatory checkbox; it is a fundamental pillar of rational drug design. We hope this Research Topic serves as both a foundational resource and a catalyst for future innovations, guiding researchers and drug developers in their shared mission to deliver safer, more efficacious, and more accessible life-changing biologics to patients worldwide.

